# Comparison between self sampling and provider collected samples for Human Papillomavirus (HPV) Deoxyribonucleic acid (DNA) testing in a Nigerian facility

**DOI:** 10.11604/pamj.2018.30.110.14321

**Published:** 2018-06-12

**Authors:** Olusegun Kayode Ajenifuja, Nzechukwu Zimuod Ikeri, Olawale Victor Adeteye, Adekunbiola Aina Banjo

**Affiliations:** 1 Department of Obstetrics and Gynecology, Obafemi Awolowo University, Ile-Ife, Nigeria; 2 Department of Anatomic and Molecular Pathology, Lagos University Teaching Hospital; 3 Department of Anatomic and Molecular Pathology, College of Medicine University of Lagos

**Keywords:** Pap smear, HPV screening, cervical cancer, sample collection, self-sampling, provider collected, PCR, HPV DNA, Ile-Ife Nigeria

## Abstract

**Introduction:**

The multiple visits required for an effective Pap smear screening program is difficult to replicate in many developing countries. This precludes early diagnosis and care for patients with cervical cancer and contributes to its high mortality in these countries. HPV screening has higher specificity and high negative predictive value and has the advantage that materials can be self-collected, which permits the screening of women who for various cultural and religious reasons would be reluctant to come to the clinic to expose themselves for screening. The aim of the study was to assess the degree of agreement between self sampling for HPV DNA with samples collected by a health provider.

**Methods:**

Each respondent selected from women presenting for cervical cancer screening underwent both self- and provider sampling for HPV DNA testing using Hybribio GenoArray.

**Results:**

Of the 194 women screened, 12 (6.2%) and 19 (9.8%) had HPV on self sampling and provider col-lected samples respectively. The commonest HPV type seen using both techniques was HPV 58 (2.6%). Multiple HPV genotypes were seen in 1 (0.5%) and 5 cases (2.6%) of provider and self-collected samples respectively. The high risk-HPV detection rate was 7.2% when self sampled and 6.8% when sampled by the provider. There was moderate correlation between both sampling techniques (κ = 0.47, 95% CI: 21.3 - 72.3%, P < 0.05).

**Conclusion:**

Our study shows moderate correlation between both sampling techniques. Larger multicentre studies will be needed to provide results generalisable to the Nigerian population. Keywords: Pap smear, HPV screening, cervical cancer, sample collection, self-sampling, provider collected, PCR, HPV DNA, Ile-Ife Nigeria.

## Introduction

Cancer of the cervix is the third leading cause of cancer deaths in women worldwide following breast and colorectal cancers. Over the past 30 years however the incidence and mortality rates have declined in developed countries. This is largely due to effective and efficient cervical cancer screening programs using pap smears and HPV DNA testing [1]. Testing for high risk HPV DNA has been shown in randomized controlled trials to offer 60-70% more protection against invasive cervical carcinomas over cytology and allows for extension of screening intervals [2, 3]. These factors make cervical cancer the most preventable gynecological cancer [4]. In recent times however there has been concerns about reduction of coverage resulting from non-attendance of screening programs in some western countries [5,6]. Reasons given include embarrassment, anticipated pain, forgetting to make appointments, lack of awareness of recommended screening interval, dislike of the health care giver and lack of time. Self sampling for HPV DNA testing provides an alternative to sampling by health care givers and indeed has been shown to improve attendance rates [5]. Its results are also comparable to those of provider collected samples depending on the sample brush or polymerase chain reaction (PCR) technique used [7]. Eighty percent of cases and 88% of deaths attributed to cervical cancer occur in low to middle income countries, with sub-Saharan Africa having the highest burden of the disease (age standardized incident rate of 50/100, 000 compared to 5/100, 000 in high income countries) [1]. Barriers to screening in developing countries include competing health needs, limited human and financial re-sources, poorly developed healthcare services, disempowerment of women, widespread poverty, war and civil strife [3]. Due to these barriers in screening, visual inspection under acetic acid (VIA) is common among these populations. Unfortunately VIA performs poorly when compared to conventional cytology and HPV DNA testing [1]. HPV DNA testing on self sampled specimen might therefore serve as a reasonable alternative for screening in low to middle income health countries because of its potential to overcome some of these barriers [3]. Its feasibility however has not been assessed in many of these countries. The aim of this study was therefore to assess the degree of agreement between self and provider collected samples for HPV DNA.

## Methods

**Study population:** The study was conducted at the Gynaecological Oncology Clinic at Obafemi Awolowo University Teaching Hospital Complex (OAUTHC) Ile-Ife, a community based clinic for screening of cervical cancer as well as other diseases of the female genital tract. Informed consent was obtained from the women and ethical clearance was obtained from the Ethical Committee of OAUTHC.

**Sample collection:** Systematic random sampling technique was used to stratify patients into two groups. Respondents in group A underwent provider sampling before self sampling, while respondents in group B had self sampling before undergoing provider sampling. All respondents underwent both sampling procedures. Both self collected samples and provider collected samples were obtained using cytobrush (cervexR) cervical cell sampler. Provider collected specimens were obtained by sampling the squamocolumnar junction using a 360° rotator movement of the cytobrush after the introduction of a speculum. The respondents were taught how to perform the procedure for sampling the upper vagina according to the instructions on the sample collection kit. Both specimens were dropped in the Hybribio HPV DNA collection kit provided for HPV DNA test.

**HPV genotyping:** The samples were frozen at -20°C and sent to the College of Medicine University of Lagos for HPV typing which was done using HPV GenoArray test kits (Hybribio Biochemical Company Limited, China) for 21 HPV genotypes which included high risk types 16, 18, 31, 33, 35, 39, 45, 51, 52, 56, 58, 59, and 68, low risk types 6, 11, 42, 43, 44, and 81 and probable high risk types 53 and 66. The assay was performed according to the manufacturer's protocol. After storage at -20°C, DNA was extracted by cell lysis and centrifugation. DNA amplification was performed in a Perkin-Elmer GeneAmp PCR system 9700 Therma cycler using 5μl of DNA template, 19.25μl of master mixture, and 0.75μl of DNA Taq polymerase. The protocol for amplification was 9 mins of denaturation at 95°C, 40 cycles of denaturation at 95°C for 20s each, 30s of annealing at 55°C, 30s of elongation at 72°C and final extension for 5 minutes at 72°C. This was done alongside positive and negative controls. After amplification the sample was denatured by heating at 95°C for 5 minutes, mixed with 0.5mls pre-warmed hybridisation solution and incubated for 20 minutes, after which a blocking solution was added. This flow-through hybridisation procedure was performed in a sample well atop a Hybrimem HPV-21 membrane containing immobilised probes against which target molecules were directed. Streptavidin-horseradish peroxidase conjugate was added to bind to the biotinylated PCR products. The direct visualisation of the breakdown product (purple precipitate) of the substrate nitroblue tetrazolium-5-bromo-4-chloro3-indolylphosphate was interpreted as positive for the corresponding HPV DNA type as indicated on the schematic diagram of the membrane provided with the test kit.

**Data analysis:** The data collected were analyzed using the Statistical Package for the Social Sciences for Windows version 22.0 (IBM, Armonk, NY, USA). The agreement between the two collection methods were calculated using concordance and discordance rates and the Cohen's kappa statistic. The crude percent agreement between the two techniques was the percentage of samples concordant or discordant for the presence of high risk HPV DNA. The kappa statistic was calculated to determine the level of chance-agreement between the two methods with a kappa value of 0 indicating no agreement better than chance, a value of 1 indicating perfect agreement better than chance, and intermediate values of 0.00-0.20, 0.21-0.40, 0.41-0.60, 0.61-0.80 and > 0.81 indicating poor, fair, moderate, good and excellent agreement respectively. P values of <0.05 were taken as statistically significant.

## Results

A total of 194 women were recruited for the study. The age range was 23-75 years (mean 43.4, SD9.6). The peak age group of respondents was 30 to 50 years, while women above 60 years accounted for the fewest (5%). The mean age of first menstrual period was 18 years, of first sexual exposure was 23.3 years, and of first pregnancy was 25.7 years. The mean parity was 4. Majority of the participants (86%) were in monogamous marriages. Most of the women in this study 76.8% had only one life time sexual partner. Twelve of the 194 (6.2%) women showed positive HPV DNA on provider sampling, while 19 of the respondents (9.8%) showed positive HPV DNA on self-sampling. Multiple HPV genotypes were seen in 1 case (0.5%) and 5 cases (2.6%) of provider collected and self collected samples respectively. The commonest HPV DNA type seen on both provider and self collected samples was HPV 58 (5 cases in each; 2.6%). All HPV types seen on provider collected samples were high-risk types, giving a high risk HPV detection rate of 6.2%. Fourteen high-risk HPV types were seen on self sampling, giving a high-risk HPV detection rate of 7.2%. When all the high risk HPV subtypes were considered as a group (group A) against non-high risk HPV/ no HPV as a second group (group B), there were 183 concordant and 11 discordant cases between both sampling methods, i.e. there was an agreement on 7 cases as having high risk HPV, 176 cases as having non-high risk/no HPV and a disagreement on 11 cases as having high risk HPV between both sampling methods. Of the 7 concordant cases, both sampling methods detected the exact same HPV subtype in 6 cases. This translated into a concordance rate of 93.8%, a discordant rate of 6.2%, and an overall moderate agreement between the two methods (κ = 0.47, 95% CI: 21.3-72.3%, P < 0.05).

## Discussion

Cancer of the cervix continues to be a public health problem in developing countries with higher age standardized incidence rates, life time risk and mortality rates than western societies. The age stand-ardized incidence rate of cervical cancer in West Africa in 2012 was reported to be 29.3/ 100,000 women. This value is high when compared to figures from western countries: 6.6/100, 000 women in the North America and 7.3/100,000 women in the Western Europe. The same is the case for life-time risk of developing cervical cancer (3.2/100 in West Africa compared to 0.7/100 in Western Europe) and the mortality rate (10.4/100, 000 in West Africa compared to 4.0/100, 000 in North America) [8]. These figures emphasize the need for an effecting cervical cancer screening program in sub-Saharan Africa. Services that help circumvent the barriers to screening are therefore welcome. The prevalence of HPV DNA in this study is low (9.8% on self-sampling and 6.2% on provider sampling) when compared to other local studies. Incidence rates of 26.3%, 14.7% and 11.34% were reported in Ibadan, Irun and Lagos respectively [9-11]. The particularly high value reported in Ibadan could be due to the greater number of HPV DNA types studied (44 HPV types compared to 21 in this study, 13 in the Irun study and 21 in the Lagos study). However in a study conducted in Gombe which tested for only 11 HPV types, a prevalence rate of 48.1% was reported [12]. Though the cases seen in that study were those referred from primary health care workers following symptoms referable to the female genital tract, a prevalence of 76% reported in Kano, also in the northern part of Nigeria, suggests a higher prevalence of HPV in northern Nigeria [13]. Early pregnancy, high parity and polygamy, which have been noted in this part of the country, are the possible explanations for the high HPV prevalence. Prevalence values of high risk HPV types have been reported as 25% in the US, 20.1% in Norway and 40% in the UK [4-6].

The detection rate of HPV also depends on the assay method used. The different genotyping tests used in various studies make comparison of results difficult. In one report, self collected vaginal samples had a sensitivity of 70.9% for ≥ CIN 3 compared to 95% for provider collected endocervical samples when assayed using cervista. The sensitivity increased to 94.3% for self collected virginal samples, which was identical to the sensitivity (94.3%) obtained on provider collected sample when the more sensitive PCR based matrix-assisted laser desorption/ionization time-of-flight (MALDI-TOF) was used [7]. In another study, 4 PCR based tests were assessed. These included three L1-PCR-based assays: PGMY09/11 LBA, HPV DNA Chip and SPF LiPA, and one E1-PCR based assay - the E1 consensus PCR followed by cycle sequencing (E1-PCR). When compared with the histology, the clinical sensitivities for detection of ≥ CIN 2 were 100% for E1-PCR, 95.2% for PGMY09/11 LBA, 100% for SPF-10 LiPA and 84.1% for the HPV DNA Chip. The concordance rates varied from poor (κ = 0.20) to intermediate (κ=0.54) for HPV detection, though this became higher for the detection of high-risk HPV types [14]. PCR based tests have also been shown to be more sensitive than testing with hybrid capture assays [15]. The most prevalent HPV DNA subtype varies in different geographical locations. In this study, HPV 58 and 39 were the commonest subtypes seen. This is in contrast to HPV 35 and 16 in Ibadan, 35 and 52 in Abuja, HPV 52 in Kenya, and HPV 16 and 66 in Chile [9, 16-18]. In a study done in China, the HPV infection rate was found to be significantly higher in women in the southwestern region (19.9%) compared to other regions (11.1% northeastern, 12.9% central). In that study HPV-52 was the most prevalent genotype in the central and northeast regions while HPV 16 was the most prevalent type seen in the northwest and southeast regions [19]. This variability is similar to another study conducted in Mexico where there was a difference in the distribution of the HPV genotypes between two regions of the southwest pacific coast [20]. These differences are most likely due to epidemiological factors and might explain the discordance between our findings and those of other parts of S.W Nigeria.

Self-sampling for HPV DNA, as an alternative to sampling by health care givers, has been well received in some studies. In one report of Hispanic respondents, the overall experience was reported as excellent by 33.7% and very good by 30.8%. Only 2.6% reported a poor of fair experience. The clarity of instructions, ease of use of kit and understanding of results were aspects of the experience the respondents found favourable [21]. A study in Norway showed that attendance rates increased from 23.2% to 33.2% when non-attenders had a choice between home-based self-sampling and appointments by a health provider. The majority of respondents in the study found the self-sampling procedure to be easy, not painful, embarrassing or scary [6]. Self sampling is also well received in Nigeria. A study done in Abuja showed a higher proportion of women in the self-collection group completing the HPV tests compared to those invited to the health facility for specimen collection by a health worker. In that study the majority of respondents found the sample device easy to use and chose self-sampling as the preferred method to be used in the future [16]. In Kenya where lack of transportation, cost and long hospital queues are reported deterrents to hospital-based screening, self-sampling has been proposed as a viable alternative for ensuring cervical screening [22]. Most studies report good concordance between self-sampling and sampling by health care providers. In this study there was moderate concordance between self-collected and provider collected samples. A good correlation was also seen in a Canadian study with concordance rate of 85.7% and kappa coefficient of 0.54 [23]. Concordance rates of 93.8% (κ = 0.76, 95% CI: 71.8-89.0%), 93.2% (κ = 0.81; 95% CI: 0.69-0.94) and 92% (κ = 0.75) have been reported in India, Seoul and Uganda respectively [24-26]. The lower kappa value in our study is most probably due to the smaller sampling size and possibly the technique of DNA retrieval. All these values show that self-sampling may be a viable screening alternative with the potential for increasing cervical cancer screening coverage and therefore reducing the prevalence of cervical cancer. The major limitation of this study is the small sample size and single centre of study. Larger multicenter studies are important for providing results that are generalisable to the Nigerian population.

## Conclusion

This study found moderate correlation between self and provider collected samples for HPV DNA detection. However due to the small sample size and single centre of study, we recommend larger multicenter studies which would provide more representative results of HPV infection rates in the Nigerian population.

### What is known about this topic

- Incidence rates of HPV in Ibadan, Irun and Lagos respectively are 26.3%, 14.7% and 11.34%;
- The commonest HPV types are 35 and 16 in Ibadan, 35 and 52 in Abuja, HPV 52 in Kenya, and HPV 16 and 66 in Chile,the overall experience of self sampling is favourable: it was reported as excellent by 33.7% and very good by 30.8% among Hispanics;
- The attendance rates for cervical screening increased from 23.2% to 33.2% in Norway when self-sampling was introduced, there is good correlation between self and provider sampling: 85.7% concordance in Canada, 93.8% in India, 93.2% in Seoul and 92% in Uganda.

### What this study adds

- The commonest HPV DNA in this study is HPV 58. This is of epidemiologic significance as it is a high risk type;
- There was a concordance of 93.8%, and a discordance of 6.2% between both sampling techniques. The concordance is high when compared with other studies;
- There was an overall moderate agreement between the two methods (κ = 0.47, 95% CI: 21.3 - 72.3%, P < 0.05).
